# Preoperative risk assessment for postoperative pancreatic fistula (POPF): Image-based calculation of duct-to-parenchyma (D/P) ratio and an Alignment of Duct and Mucosa (ADAM) anastomosis may lead to a low POPF rate—results from 386 patients

**DOI:** 10.3389/fsurg.2022.1039191

**Published:** 2022-11-11

**Authors:** Colin M. Krueger, Melanie Langheinrich, Esther A. Biesel, Lena Kundel, Karsten Krueger, Ulrich Adam, Hartwig Riediger

**Affiliations:** ^1^ Department of Surgery, Immanuel Clinic Ruedersdorf, University Clinic of Brandenburg Medical School, Berlin, Germany; ^2^ Department of Surgery, Clinic of General-, Visceral-, Vascular and Thoracic Surgery, University Medicine Greifswald, Greifswald, Germany; ^3^ Department of General and Visceral Surgery, Faculty of Medicine, Medical Center—University of Freiburg, Freiburg im Breisgau, Germany; ^4^ Department of General Surgery, Vivantes-Humboldt Hospital, Berlin, Germany; ^5^ Institute of Diagnostic and Interventional Radiology, Vivantes-Humboldt Hospital, Berlin, Germany

**Keywords:** pancreatic surgery, postoperative pancreatic fistula, alignment of duct-and-mucosa, pancreatojejunostomy, preoperative risk assessment

## Abstract

**Background:**

Postoperative pancreatic fistula (POPF) is the most critical complication after pancreatoduodenectomy (PD). Preoperative identification of high-risk patients and optimal pancreatic reconstruction technique can be a way to reduce postoperative complications.

**Methods:**

A series of 386 patients underwent PD over a 10-year period (2009–2019). On routinely performed preoperative computed tomography (CT) images, the ventro-dorsal diameters of duct (D) and parenchyma (P) were measured in the cutting plane at the superior mesenteric vein. Then, the ratio of both values was calculated (D/P ratio) Double-layer pancreatojejunostomy with alignment of duct and mucosa (ADAM) by two monofilament threads (MFT) was performed in 359 patients and pancreatogastrostomy (PG) in 27 patients. The incidence of POPF was diagnosed according to the International Study Group for Pancreatic Fistula criteria.

**Results:**

The overall rate of POPF was 21% (*n* = 80), and the rate of clinically relevant type B/C fistulas 6.5% (*n* = 25). A D/P ratio of <0.2 was significantly associated with type B/C fistula (11%, *p* < 0.01). In low-risk patients (D/P ratio >0.2), type B/C fistula occurred only in 2%, and in high-risk patients (D/P ratio <0.2) in 9%. ADAM anastomosis was performed safely by two different surgeons. A PG anastomosis had double-digit POPF rates in all groups.

**Conclusion:**

Preoperative CT imaging with D/P measurement may predict the risk of POPF development. A cut off D/P ratio of <0.2 was significantly associated with clinical relevant POPF. ADAM anastomosis may be an option for pancreatojejunostomy. However, preoperative knowledge of the D/P ratio could guide decision-making for primary pancreatectomy when pancreatic reconstruction is critical.

## Introduction

Pancreatic fistula is one of the most severe complications after pancreatoduodenectomy and a major cause of morbidity and mortality in pancreatic surgery. Despite all advantages in surgical technique and perioperative management, in-hospital mortality varied from 6.5% (high volume hospitals) to 11.5% (low volume hospitals) in Germany (1).

A universally accepted definition and grading of postoperative pancreatic fistula has been developed in 2005 from the International Study Group of Pancreatic Fistula (ISGPF). In 2016 the ISGPF revised the definition and introduced new criteria (2, 3). Thus, POPF can be divided in two major groups: clinically irrelevant (i.e., biochemical leak) and clinically relevant fistula requiring further therapy (POPF B and C). A current transatlantic comparison of 4 large registries from the United States, Germany, Sweden, and Netherlands with 22,983 pancreatoduodenectomies yield a clinically relevant pancreatic fistula in up to 16% (4). Many factors have been associated with pancreatic fistula, including patient related factors (such as body mass index, diameter of the pancreatic duct, pancreatic texture), surgical technique and experience (5).

To prevent pancreatic fistula, various technical procedures have been described including the invaginating pancreatojejunostomy, the duct-to-mucosa pancreatojejunostomy with several modifications, the Blumgart anastomosis and pancreatogastrostomy (PG) (6, 7). So far, no technique is superior to the other (8). Advantages were attributed to PG in difficult anastomotic conditions. Nevertheless, the duct-to-mucosa pancreatojejunostomy is one of the most used techniques in clinical practice. Despite the large number of publications, POPF still remains a major challenge for surgeons and a risk adapted approach is recommended to achieve optimal results (9).

**Figure 1:**
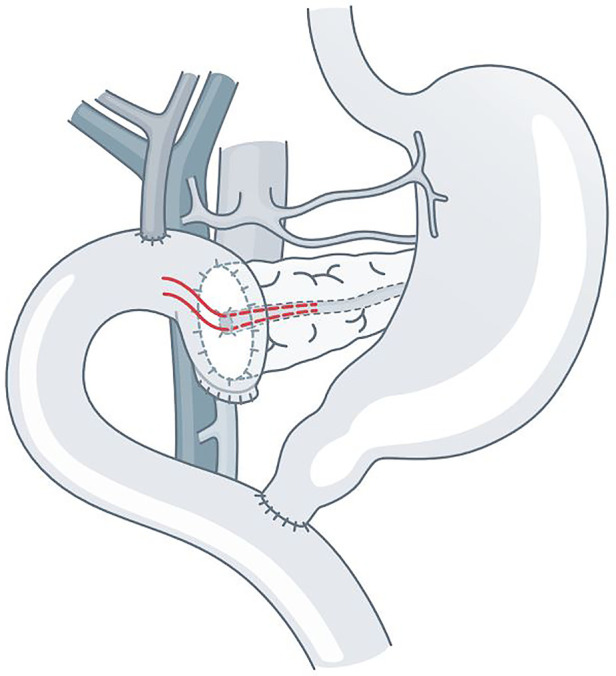
ADAM anastomosis with two monofilament threads (red) (MFT).

Here we report our results of a modified pancreatojejunostomy technique performed in our center over a period of more than 15 years. The two-layer duct-to-mucosa anastomosis was performed with a non-tubular internal splint. We refer to it as ADAM anastomosis (Alignment of Duct And Mucosa), with two absorbable MFT (Figure 1).

In a previous study (2006–2010, *n* = 139) we were able to show that ADAM is safe (10). Theoretically, by the precise placement of two MFT, they should provide protection for the PJ. The shown technique was already the technical standard for reconstruction in pancreatic head resection at the beginning of our study period. Thereby allowing the pancreatic juice to be directly drained and thus reducing arrosion at the anastomotic site in the early postoperative phase until the anastomosis heals. Several studies analyzed the benefit of tubular pancreatic duct stenting, external or internal, with inconclusive results (11–13).

**Figure 2:**
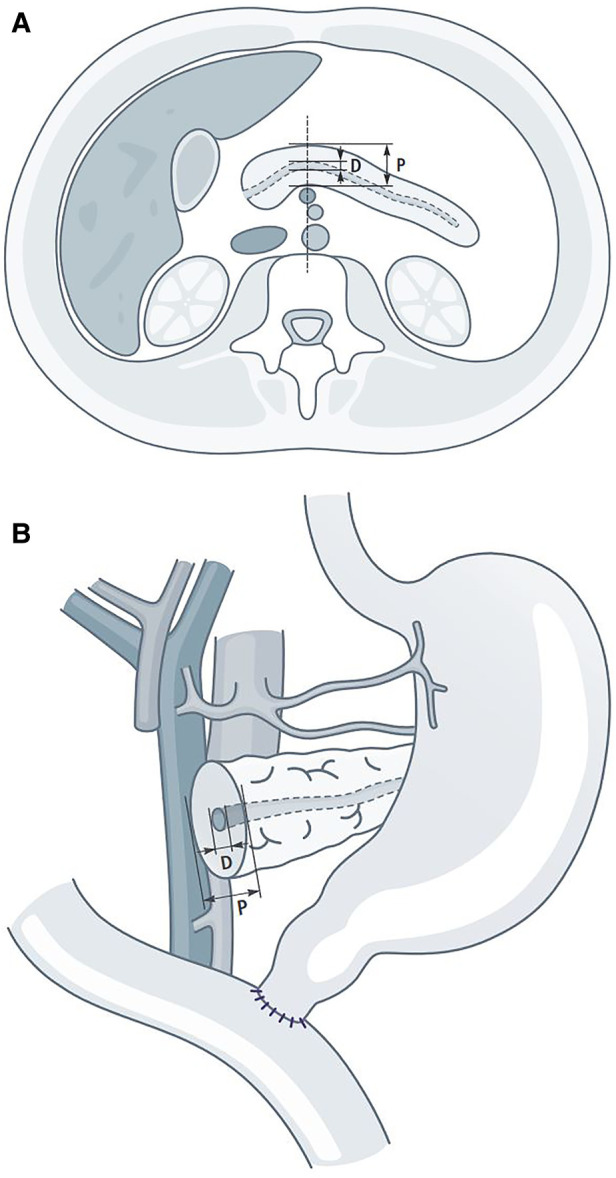
D/P ratio: (**A**) radiological D/P ratio. (**B**) anatomical D/P ratio (surgical view).

However, in another study (2009–2013, *n* = 123), a CT based risk evaluation for the occurrence of postoperative pancreatic fistulas was analyzed (14). As mentioned before, one of the most important factors related to pancreatic fistula is the diameter of the main pancreatic duct. We measured the diameter of duct (D) and parenchyma (P) at the expected cutting plane on routine preoperative CT images and calculated the quotient of both (D/P ratio). We were able to show, that a cut off value of <0.2 for the D/P ratio, when measured in the ventro-dorsal plane (Figure 2), was significantly associated with the occurrence of POPF.

The aim of the present study was to further validate clinical results of the ADAM anastomosis in context with the D/P-ratio.

## Materials and methods

This study examines a series of 386 consecutive pancreatic head resections at one hospital over a 10-year period (2009–2019) in which pancreatic reconstruction was performed using ADAM or PG. Data was prospectively collected and retrospectively evaluated. Surgery was performed by two surgeons, surgeon A with 20 years of experience in pancreatic surgery (PS) and surgeon B with 10 years of experience in PS.

### Image (CT) based evaluation of the duct to parenchyma ratio (D/P-ratio)

To determine the D/P ratio for this study, the ventro-dorsal axis was measured at the level of the confluence of the mesenteric and lienal veins, in the sense of the later transection line on the pancreas. A preoperative CT was available in 358 cases (358/386: 93%) for current evaluation in the digital hospital archive, thus the number of patients varies in Tables 2–4 due to the fact, that CT images were not available in all cases. Since the D/P ratio was only evaluated for this study, it played no role for the intraoperative choice of anastomotic technique (Figure 2). Based on literature data, patients with a D/P ratio >0.2 are classified as low-risk and those with a D/P ratio <0.2 are classified as high-risk patients for the occurrence of POPF.

### Type of pancreatic reconstruction

In the present study, the ADAM anastomosis represents the surgical standard in reconstruction. Since—at the surgeon's discretion—the PG was the preferred alternative technique in difficult anastomotic situations. The ADAM anastomosis and the PG anastomosis were comparatively examined in this work.

In both techniques, the reconstruction phase begins with the pancreatic anastomosis, followed by the creation of the biliodigestive anastomosis. Finally, the reconnection of the post-pyloric stomach is performed.

The ADAM anastomosis (ADAM) is a two-row pancreatojejunostomy with an additional internal alignment of the duct-to-mucosa anastomosis. It is made as an end-to-side anastomosis. All suture rows are presented as single button suture. Both suture rows are made with monofilament, absorbable suture lines of strength 5/0. For duct and mucosa alignment, two MFT are placed before the closure of the anterior duct-to-mucosa suture row (Figure 1).

Pancreatogastrostomy is done by performing an anterior and posterior gastrostomy. Through the posterior mini gastrostomy, the pancreatic corpus is pulled approximately 2 cm into the gastric lumen. A purse string suture and circular single button sutures of strength 4/0 are then performed between the stomach wall and the pancreatic capsule *via* the anterior approach. Finally, the anterior gastrostomy is closed with continuous absorbable closure. Local drains are placed at the end of the surgery.

### Evaluation of POPF

Postoperatively, drains amylase values were measured at days 1 and 3 and evaluated in clinical practice according to the ISGPF classification system. The classification of POPF, in terms of the current classification of 2016, was done retrospectively for the data presented 2009–2019.

### Data processing

All clinical data on pancreatic surgery were recorded in a local database (IBM SPSS 23.0). For statistical analysis, frequencies were determined, and cross-tabulations were used to calculate significance. The significance criterion was defined as *p* < 0.05 in the Fisher exact test. The anonymized data analysis was authorized by a vote of the local ethics committee [Ethics Committee of the Ärztekammer Berlin (Eth-10-21)].

## Results

### Patient characteristics

In the study period from 2009 to 2019, a total of 386 patients underwent pancreatic head resection. A pylorus-preserving pancreatic head resection (PPPD) was performed in 328 patients (85%), and a classic Whipple operation (Whipple) was performed in 58 patients (15%). Pancreatic anastomosis was performed as ADAM anastomosis in 359 cases (93%) and pancreatogastrostomy (PG) in 27 patients (7%). The procedures were performed by surgeon A (*n* = 266; 69%), surgeon B (*n* = 117; 30%) and others (*n* = 3; 1%) (Table 1).

**Table 1:** Univariate analyses of factors related to clinically relevant postoperative pancreatic fistula type B/C.

	Clinical relevant type B/C fistula	*p*
All patients (*n* = 386)	25/386 (6%)	
ADAM anastomosis (*n* = 359)	19/359 (5.3%)	
Pancreatogastrostomy (*n* = 27)	6/27 (22%)	
Age (≥80 years) (*n* = 49)	3/49 (6%)	0.62
BMI >25 kg/m^2^ (*n* = 196)	16/196 (8%)	0.12
Preoperative biliary drainage (*n* = 194)	14/194 (7%)	0.35
Malignancy (*n* = 283)	16/283 (6%)	0.19
Chronic pancreatitis (*n* = 55)	3/55 (6%)	0.51
Cystic neoplasia (*n* = 34)	4/34 (12%)	0.17
Surgeon A (*n* = 269)	17/269 (6%)	0.50
Surgeon B (*n* = 114)	7/114 (6%)	0.53
D/P-ratio <0.2 (*n* = 161/358)^a^	18/161 (11%)	<0.01

^a^ CT Abdomen with data available in 358 cases.

Patients characteristics are shown in Table 1. Indications for surgery were malignancies (*n* = 283; 73%), chronic pancreatitis (*n* = 55; 14%), cystic neoplasms (*n* = 34; 9%) and others (*n* = 14; 4%). Morbidity was (*n* = 226) 59%, and in-hospital mortality was (*n* = 13) 3.4%.

A postoperative pancreatic fistula occurred in 80 patients (20.7%). A biochemical leak was detected in 55 patients (14.2%). A clinically relevant fistula was observed in 16 patients (Type B) and 9 patients (Type C), summarized as Type B/C fistula (*n* = 25; 6.5%).

### D/P-ratio and POPF risk

A D/P-ratio of <0.2 was univariate significantly associated with POPF development. In this subgroup (D/P-ratio <0.2; *n* = 161), POPF occurred in 57 (35%) patients. A biochemical leak occurred in 39 (24%) patients and 18 patients (11%) had a clinically relevant type B/C fistula (Table 2).

**Table 2:** Preoperatively estimated D/P ratio <0.2 correlates significantly with the risk of POPF development.

	D/P-ratio <0.2 (*n* = 161)	D/P-ratio >0.2 (*n* = 197)	*p*
All pancreatic fistulas (*n* = 76)	58 (35.4%)	19 (9.6%)	<0.01
Biochem leak (*n* = 54)	39 (24.2%)	15 (7.6%)	<0.01
B/C fistulas (*n* = 22)	18 (11.2%)	4 (2%)	<0.01

### ADAM anastomosis and POPF risk

Postoperative pancreatic fistulas occurred in (*n* = 84; 17.8%) with ADAM anastomosis (*n* = 359). A biochemical leak was detected in 45 patients (12.5%). Clinically relevant type B/C were observed in (*n* = 19; 5.3%). PG was performed more frequently in patients with D/P-ratio <0.2. Here, POPF occurred in (*n* = 11; 55%). Of these, biochemical leak was present in 6 patients (30%), and clinically relevant type B/C were observed in (*n* = 5; 25%) (Table 3).

**Table 3:** Comparison of ADAM anastomosis vs. pancreatogastrostomy in relation to D/P ratio.

	ADAM Anastomosis (*n* = 331)	Pancreatogastrostomy (*n* = 27)	*p*
**D/P-ratio >0.2 (*n* = 197)**	***n* = 190**	***n* = 7**	** **
Pancreatic fistulas (*n* = 19)	14/190 (7%)	5/7 (71%)	<0.01
Biochem leak (*n* = 15)	11/190 (6%)	4/7 (57%)	<0.01
B/C fistulas (*n* = 4)	3/190 (2%)	1/7 (14%)	<0.05
**D/P-ratio <0.2 (*n* = 161)**	***n* = 141**	***n* = 20**	** **
Pancreatic fistulas (*n* = 57)	46/141 (33%)	11/20 (55%)	<0.05
Biochem leak (*n* = 39)	33/141 (23%)	6/20 (30%)	0.35
B/C fistulas (*n* = 14)	13/141 (9%)	5/20 (25%)	0.05

In the low-risk situation, ADAM anastomosis shows excellent results with a rate of 2% of clinically relevant type B/C fistulas. Compared to ADAM anastomosis, the PG anastomosis shows a significant increase in POPF, even in patients classified as low-risk. In the high-risk situation, a clinically relevant POPF (B/C fistula) occurred in 9% of patients from the ADAM group. In contrast, the pancreatogastrostomy group, yielded a type B/C fistula rate of 25%.

### Surgeon volume

Surgeon A performed more than double the number of procedures presented compared to Surgeon B. Interestingly, Surgeon A (101/245: 41%) performed procedures from the D/P-ratio <0.2 group, but Surgeon B did so in proportion (59/110: 53%). The data presented here show that the ADAM anastomosis can be performed with a high degree of safety even by less experienced surgeons. Especially in low-risk patients, a very low rate of POPF can be achieved. In high-risk patients, there is no significant difference between the two surgeons (Table 4).

**Table 4:** Risk of POPF for ADAM anastomosis comparing two surgeons (*n* = 355).

	Surgeon A (*n* = 245)	Surgeon B (*n* = 110)	*p*
**D/P-ratio >0.2 (*n* = 195)**	***n* = 144**	***n* = 51**	** **
All pancreatic fistulas (*n* = 18)	17 (12%)	1 (2%)	0.03
Biochem leak (*n* = 14)	14 (10%)	0 (0%)	0.01
B/C fistulas (*n* = 4)	3 (2%)	1 (2%)	0.72
**D/P-ratio <0.2 (*n* = 160)**	**(*n* = 101)**	**(*n* = 59)**	** **
All pancreatic fistulas (*n* = 56)	34 (34%)	22 (37%)	0.38
Biochem leak (*n* = 39)	22 (22%)	17 (28%)	0.20
B/C fistulas (*n* = 17)	12 (12%)	5 (9%)	0.35

## Discussion

To assess the risk of POPF, the diameter of the duct and organ strength have been established (15–18). The assessment is undoubtedly best done intraoperatively. However, a risk assessment in the preoperative planning phase would be advantageous. Today, indirect indicators for POPF can be obtained from diagnostic imaging. For example, density measurements of the parenchymal organs or volumetry of the residual pancreas have proven suitable (19, 20). The procedure, we have described here, can be performed on any routine CT, even without detailed radiological expertise. The risk assessment is based on two easy to collect measurements (14) (Figure 2).

The idea for the resorbable alignment technique, described here, arose in the typical situation of difficult pancreatic organ situation, which are essentially characterized by a delicate duct and soft parenchyma and thus weak suture bed. Non-absorbable material can lead to migration and tissue damage even after years (21). An external drainage requires an additional jejunostomy. For this reason, the described variant of a doubled MFT made of absorbable material is, in the authors' opinion, an optimal solution.

Pancreatic anastomosis fistulas are considered a trigger for specific complications such as gastric emptying disorders and arterial bleeding (22, 23). Surgical expertise has a strong influence on perioperative outcome (24). As recently published, an increased risk of POPF has been described when anastomosis is performed by less experienced surgeons in very tender Wirsingianus duct (25). This particular risk must be addressed by a simple and reproducible surgical approach.

During the 10-year study period, the more experienced surgeon A performed more than twice as many operations as surgeon B. However, surgeon B operated on more patients with the risk factor D/P-ratio <0.2. One possible explanation is that the complexity of the resection was the primary criterion in the team planning for surgery. In our experience, patients with a dilated duct often have more difficult local findings if the resection alone is focused on. In perspective, it seems reasonable to evaluate the severity of resection and reconstruction separately. The preoperative D/P ratio can thus be a criterion for team planning in the resection and reconstruction phase.

Reconstruction can be much more complex in patients with a delicate gait.

Nevertheless, there was no difference in clinically relevant B/C fistulas in both operators, which speaks for a high degree of standardization and reproducibility of this anastomosis technique.

For the large number of low-risk patients, there is a low rate of POPF in the overall ADAM anastomosis group. This is true for the biochemical leak and the clinically relevant B/C fistulas. For high-risk patients, the results are less clear. The individual case decisions on PG did not provide an advantage in the presented patient group. A comparative evaluation related to other anastomotic techniques cannot be performed in the present study design. Based on the large number of cases in the investigated patient group, a safe feasibility with a low POPF rate can be derived for the ADAM anastomosis.

In the future, knowledge of the D/P ratio may be even a decision-making aid for primary pancreatectomy in difficult intraoperative pancreatic parenchymal ratios (26). Especially in oncological patients, the perioperative risk must be kept low, as the timely start of multimodal postoperative therapy is crucial for long-term survival.

## Conclusion

The ADAM anastomosis presented here has proven to be a viable option in a large consecutive patient population The likelihood of POPF can be estimated using preoperative CT imaging and a simple measurement procedure. Patients with a D/P ratio <0.2 are considered at a high risk for POPF.

## Data Availability

The raw data supporting the conclusions of this article will be made available by the authors, without undue reservation.
